# P-2146. Comparison of Diagnostic Performance between Induced Sputum or Tracheal Suction and Bronchoalveolar Lavage Fluid for Diagnosis of *Pneumocystis jirovecii* Pneumonia Using Quantitative PCR

**DOI:** 10.1093/ofid/ofae631.2300

**Published:** 2025-01-29

**Authors:** Peewara Thampanyawat, Patsharaporn T Sarasombath, Anupop Jitmuang, Kamonthip Kunwipakorn

**Affiliations:** Faculty of Medicine Siriraj Hospital, Mahidol University, Bangkok, Krung Thep, Thailand; Faculty of Medicine Siriraj Hospital, Mahidol University, Bangkok, Krung Thep, Thailand; Faculty of Medicine Siriraj Hospital, Bangkok Noi, Krung Thep, Thailand; Faculty of Medicine Siriraj Hospital, Mahidol University, Bangkok, Krung Thep, Thailand

## Abstract

**Background:**

The prevalence of Pneumocystis pneumonia (PCP), primarily affecting immunocompromised individuals, notably those without HIV infection, has been increasing. Diagnosing PCP is challenging due to the lack of a standard definition. Its diagnosis mainly relies on clinical history, chest imaging, and treatment response. Conventional diagnostic methods for PCP have shown limitations, particularly in sensitivity, leading to delayed diagnoses and higher mortality rates, especially in non-HIV patients. Molecular diagnostic techniques like Polymerase Chain Reaction (PCR) offer enhanced sensitivity, speed, and accuracy. This study aims to determine the diagnostic performance of our in-house qPCR to distinguish between PCP and non-PCP cases.

Criteria used for diagnosis PCP

**Figure:**
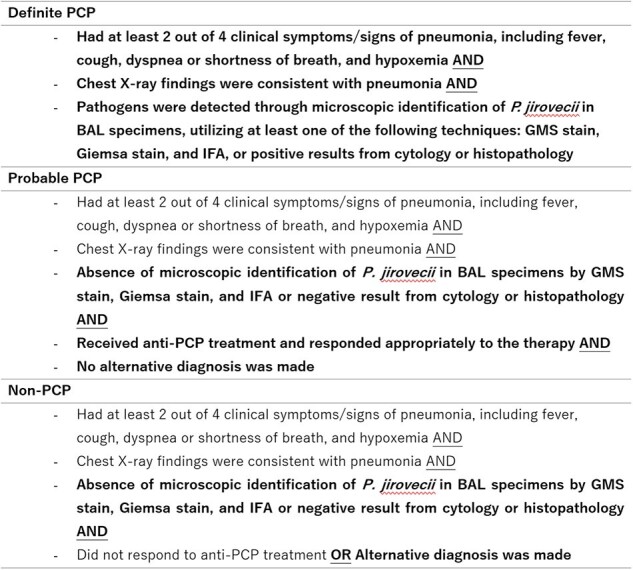

**Methods:**

A prospective study was conducted from 2022 to 2024. Immunocompromised patients with pneumonia who underwent bronchoalveolar lavage and could provide induced sputum or tracheal suction samples were included in the study. Fungal burdens in non-invasive specimens (induced sputum and tracheal suction fluid) and in invasive bronchoalveolar lavage fluid (BALF) specimens were assessed by our in-house qPCR targeting mitochondrial 12s rRNA (mt 12s rRNA). The diagnostic performance of this method for both non-invasive and invasive specimens was measured in comparison to the diagnostic criteria set for PCP. In addition, the correlation between non-invasive and invasive specimens collected at approximately the same time was compared.

Image 1: diagnostic performance of BAL qPCR for PCP

**Figure:**
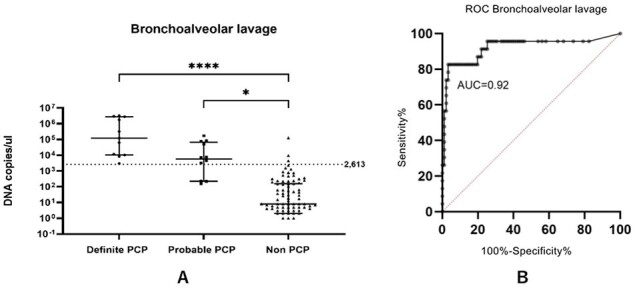

(A) Scatter plot showing P. jirovecii mtLSU-rRNA DNA copies/μl among definite PCP, probable PCP and non-PCP, Dot line: cut off value of 2,613 DNA copies/µl (B) ROC curve analysis of the BALF for the diagnosis of PCP *P-value <0.05, ****P-value <0.0001

**Results:**

Of 114 enrolled patients, 23 met PCP criteria. BAL qPCR demonstrated high sensitivity (82.6%) and specificity (96.7%), while non-invasive samples showed lower sensitivity (65.2%) and good specificity (89.0%). A BAL qPCR cutoff of 2,613 DNA copies/μl achieved high diagnostic accuracy. Tracheal suction qPCR showed a stronger correlation with BAL qPCR than induced sputum.

Image 2: diagnostic performance of sputum qPCR for PCP

**Figure:**
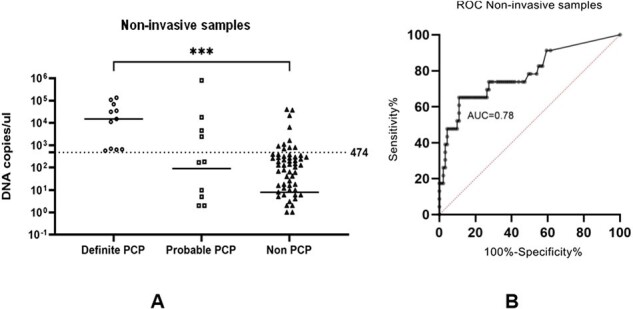

(A) Scatter plot showing P. jirovecii mtLSU-rRNA DNA copies/μl among definite PCP, probable PCP and non-PCP, Dot line: cut off value of 2,613 DNA copies/µl (B) ROC curve analysis of the non-invasive samples for PCP diagnosis., *P-value <0.05, ****P-value <0.0001

**Conclusion:**

Quantitative PCR (mtLSU-rRNA), especially from BAL fluid, enhances PCP diagnosis accuracy compared to traditional methods. Non-invasive sample qPCR remains valuable, with tracheal suction potentially preferred. These findings advance PCP diagnostic precision in immunocompromised patients, aiding prompt intervention.

Image 3: Correlation between induced sputum/tracheal suction and BALF

**Figure:**
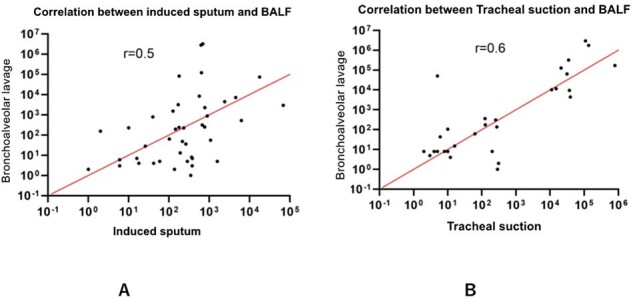

(A) Spearman's rank correlation between induced sputum and BALF (B) and tracheal suction and BALF

**Disclosures:**

All Authors: No reported disclosures

